# Redefining the Foreign Antigen and Self-Driven Memory CD4^+^ T-Cell Compartments *via* Transcriptomic, Phenotypic, and Functional Analyses

**DOI:** 10.3389/fimmu.2022.870542

**Published:** 2022-05-30

**Authors:** Takeshi Kawabe, Thomas Ciucci, Kwang Soon Kim, Shunichi Tayama, Akihisa Kawajiri, Takumi Suzuki, Riou Tanaka, Naoto Ishii, Dragana Jankovic, Jinfang Zhu, Jonathan Sprent, Rémy Bosselut, Alan Sher

**Affiliations:** ^1^ Department of Microbiology and Immunology, Tohoku University Graduate School of Medicine, Sendai, Japan; ^2^ Immunobiology Section, Laboratory of Parasitic Diseases, National Institute of Allergy and Infectious Diseases, National Institutes of Health, Bethesda, MD, United States; ^3^ Laboratory of Immune Cell Biology, Center for Cancer Research, National Cancer Institute, National Institutes of Health, Bethesda, MD, United States; ^4^ David H. Smith Center for Vaccine Biology and Immunology, Department of Microbiology and Immunology, University of Rochester, Rochester, NY, United States; ^5^ Department of Integrative Biosciences and Biotechnology, Pohang University of Science and Technology, Pohang, South Korea; ^6^ Molecular and Cellular Immunoregulation Section, Laboratory of Immune System Biology, National Institute of Allergy and Infectious Diseases, National Institutes of Health, Bethesda, MD, United States; ^7^ Immunology Division, Garvan Institute of Medical Research, Darlinghurst, NSW, Australia; ^8^ St. Vincent’s Clinical School, University of New South Wales, Sydney, NSW, Australia

**Keywords:** CD4^+^ T lymphocytes, memory, homeostasis, innate immunity, phenotypic analysis

## Abstract

Under steady-state conditions, conventional CD4^+^ T lymphocytes are classically divided into naïve (CD44^lo^ CD62L^hi^) and memory (CD44^hi^ CD62L^lo^) cell compartments. While the latter population is presumed to comprise a mixture of distinct subpopulations of explicit foreign antigen (Ag)-specific “authentic” memory and foreign Ag-independent memory-phenotype (MP) cells, phenotypic markers differentially expressed in these two cell types have yet to be identified. Moreover, while MP cells themselves have been previously described as heterogeneous, it is unknown whether they consist of distinct subsets defined by marker expression. In this study, we demonstrate using combined single-cell RNA sequencing and flow cytometric approaches that self-driven MP CD4^+^ T lymphocytes are divided into CD127^hi^ Sca1^lo^, CD127^hi^ Sca1^hi^, CD127^lo^ Sca1^hi^, and CD127^lo^ Sca1^lo^ subpopulations that are Bcl2^lo^, while foreign Ag-specific memory cells are CD127^hi^ Sca1^hi^ Bcl2^hi^. We further show that among the four MP subsets, CD127^hi^ Sca1^hi^ lymphocytes represent the most mature and cell division-experienced subpopulation derived from peripheral naïve precursors. Finally, we provide evidence arguing that this MP subpopulation exerts the highest responsiveness to Th1-differentiating cytokines and can induce colitis. Together, our findings define MP CD4^+^ T lymphocytes as a unique, self-driven population consisting of distinct subsets that differ from conventional foreign Ag-specific memory cells in marker expression and establish functional relevance for the mature subset of CD127^hi^ Sca1^hi^ MP cells.

## Introduction

Conventional CD4^+^ T lymphocytes are classically divided into two main compartments in the steady state: naïve (CD44^lo^ CD62L^hi^) and memory (CD44^hi^ CD62L^lo^) cells. The latter cell population is thought to comprise a mixture of “authentic” memory cells derived from naïve T lymphocytes responding to explicit stimulation with foreign antigens (Ags), together with cells of a similar phenotype that are formed from naïve precursors independently of foreign Ag recognition (1–3). While the function of these foreign Ag-independent “memory-phenotype (MP)” T cells is not fully understood, we have recently shown that they are able to exert innate immune function. Thus, MP CD4^+^ T cells can contribute to host defense against *Toxoplasma* infection by producing IFN-γ in response to IL-12 in the absence of Ag recognition (4, 5). Based on these findings, we proposed that together with their CD8^+^ counterparts [referred to as virtual memory (T_VM_) cells], CD4^+^ MP T cells are participants in the lymphocyte-mediated innate immunity known to be provided by natural killer (NK) and innate lymphoid cells as well as unconventional T lymphocytes such as NKT and mucosal-associated invariant T cells (6–10).

Because of the abovementioned phenotypic similarities between CD4^+^ MP and foreign Ag-specific memory cells, MP T lymphocytes were initially presumed to represent memory cells specific for foreign Ags derived from commensal microflora and/or food (1–3). Moreover, while previous studies attempted to define signals that are essential for the maintenance and survival of MP cells (11–13), most of the findings on the properties of these cells have proven to be equally applicable to foreign Ag-specific memory cells (12, 14, 15). Hence, the question of whether MP cells do indeed represent a phenotypically distinct cell population has remained unclear.

Nonetheless, there is accumulating evidence suggesting that MP and foreign Ag-specific memory cells arise from different developmental pathways. Traditionally, the generation of CD4^+^ MP cells was studied using lymphopenic animals such as irradiated or gene-manipulated mice (16–20). When naïve T lymphocytes are transferred to such animals, a few clones can generate robust proliferative responses as a result of homeostatic proliferation and acquire a memory phenotype even in the absence of explicit foreign Ag recognition. More recently, we showed that this homeostatic expansion can be driven in physiologic, lymphoreplete conditions as well (4). Thus, when transferred to lymphosufficient hosts, some naïve cells proliferate to generate a CD44^hi^ CD62L^lo^ phenotype in an Ag-recognition- and CD28-dependent fashion. Because these MP cells are equally present in unimmunized specific pathogen-free (SPF) and germ-free (GF) mice (4), self Ags are thought to be the major stimulus for their steady-state development as opposed to foreign Ags that induce conventional memory T lymphocytes. Moreover, once generated, MP cells further differentiate into an innate T-bet^+^ subset in the presence of IL-12 tonically produced by type 1 dendritic cells (5). Because this cytokine production occurs in the absence of foreign agonist-derived stimuli, T-bet^+^ MP differentiation is considered to be a self-dependent process, unlike conventional Th1 development where foreign agonist-induced IL-12 plays a critical role (21).

In addition to being expanded by different agonists, MP and foreign Ag-specific memory T cells are known to be maintained differently once generated. Thus, in a lymphopenic environment, MP cells display two different types of homeostatic proliferation referred to as slow and fast cell division, while foreign Ag-specific memory T lymphocytes exhibit a homogeneous mild rate of proliferation (12). This suggests the involvement of distinct mechanisms for MP maintenance. Under more physiologic, lymphosufficient conditions, foreign Ag-specific memory T lymphocytes are known to be quiescent (15, 22). By contrast, more than 30% of MP cells are in the cell cycle at any given time point during homeostasis (22), and during a 4-week period, ~60% have divided at least once (23). These observations suggest that MP T lymphocytes in the steady state are maintained as two different (rapidly expanding and more quiescent) subpopulations in contrast to foreign Ag-specific memory cells that divide infrequently, again supporting the concept that MP cells are qualitatively distinct.

In the present study, we have employed transcriptomic and phenotypic analyses to address the unresolved issue of whether MP and foreign Ag-specific memory CD4^+^ T lymphocytes are distinguishable from each other. Our data identify CD127, Sca1, and Bcl2 as key markers differentially expressed in these two cell populations and demonstrate the use of these markers in defining a previously unappreciated functional heterogeneity within the MP population.

## Results

### Memory-Phenotype CD4^+^ T Lymphocytes Consist of Four Subpopulations Based on CD127 and Sca1 Expression, While Foreign Antigen-Specific Memory Cells Are All CD127^hi^ Sca1^hi^

**Figure 1:**
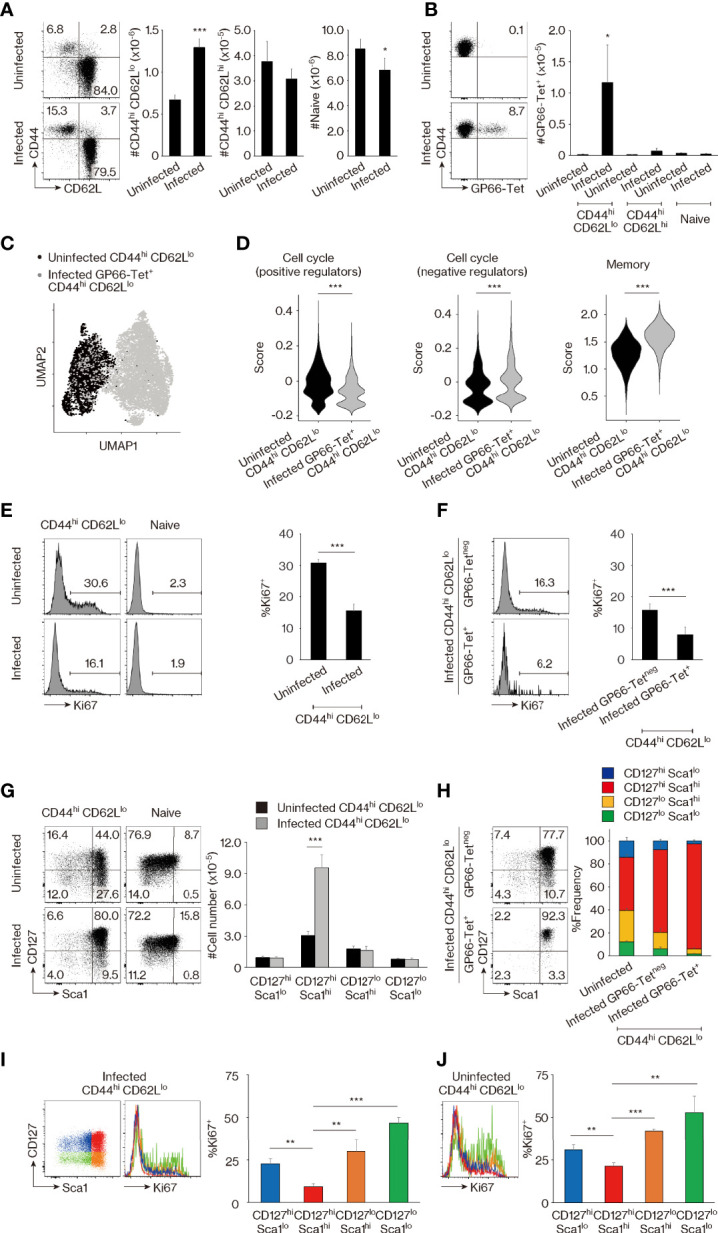
CD44^hi^ CD62L^lo^ CD4^+^ T lymphocytes represent a mixture of memory-phenotype (MP) and foreign antigen (Ag)-specific memory cells. **(A, B)** CD44^hi^ CD62L^lo^ CD4^+^ T-cell population size is larger in lymphocytic choriomeningitis virus (LCMV)-infected versus uninfected mice because of the presence of foreign Ag-specific memory cells. **(A)** The representative dot plots show CD44 and CD62L expression in Foxp3^neg^ CD4^+^ T lymphocytes from uninfected and infected animals, while the bar graphs indicate the number (mean ± SD) of CD44^hi^ CD62L^lo^, CD44^hi^ CD62L^hi^, and CD44^lo^ CD62L^hi^ (naïve) cells in the same T-cell population (n = 5 mice). **(B)** Dot plots displaying GP66-tetramer binding in CD44^hi^ CD62L^lo^ CD4^+^ T lymphocytes from each group as well as a bar graph indicating the number (mean ± SD) of GP66-tetramer^+^ cells in the indicated cell populations are shown (n = 5 mice). Data are representative of 3 independent experiments performed. **(C, D)** Comparison of MP versus foreign Ag-specific memory T lymphocytes by single-cell RNA sequencing (scRNAseq) analysis. **(C)** The plot displays single cells determined by the Uniform Manifold Approximation and Projection (UMAP) algorithm. Each dot represents a cell. **(D)** The violin plots show relative expression of genes from the indicated signatures across populations. The gene list for each signature is provided in *Materials and Methods* and **Table S1**. **(E, F)** Ki67 expression is high and low in MP and foreign Ag-specific memory cells, respectively. The histograms display Ki67 expression in the indicated cell populations, while the bar graphs indicate the frequency (mean ± SD) of Ki67^+^ cells among each population (n = 3 mice). Data shown are representative of 3 independent experiments performed. **(G, H)** MP cells consist of CD127^hi^ Sca1^lo^, CD127^hi^ Sca1^hi^, CD127^lo^ Sca1^hi^, and CD127^lo^ Sca1^lo^ subpopulations, while foreign Ag-specific memory cells are all CD127^hi^ Sca1^hi^. Representative dot plots display CD127 and Sca1 expression in the indicated cell populations, while the bar graphs show **(G)** the number (mean ± SD) and **(H)** the frequency (mean ± SD) of each cell subpopulation (n = 5 mice). Data are representative of 2 independent experiments. **(I, J)** Ki67 levels are the lowest in the CD127^hi^ Sca1^hi^ cell subpopulation in both infected and uninfected mice. Bar graphs depicting the frequency (mean ± SD) of Ki67^+^ cells among the indicated cell subsets from **(I)** infected and **(J)** uninfected animals are shown (n = 3 mice). Representative histograms displaying Ki67 expression are also included. Data are representative of 2 independent experiments. Statistically significant differences are indicated as *p < 0.05, **p < 0.01, ***p < 0.001.

In the case of CD8^+^ T lymphocytes, the population of CD44^hi^ CD62L^lo^ but not CD44^hi^ CD62L^hi^ cells expands in response to immunization with foreign Ags (24). Indeed, the former cell compartment is large in feral mice while small in animals housed under SPF conditions (25). As a first step in comparing MP CD4^+^ T lymphocytes with foreign Ag-driven memory cells, we wished to determine if a similar foreign Ag-driven expansion of the CD44^hi^ CD62L^lo^ population occurs in CD4^+^ T cells as well. To do so, we infected SPF C57BL/6 mice with lymphocytic choriomeningitis virus (LCMV) Armstrong and waited for 6 weeks to generate foreign Ag-specific memory cells defined by tetramer staining. The CD44^hi^ CD62L^lo^ but not CD44^hi^ CD62L^hi^ CD4^+^ T-cell fraction was found to be significantly larger in infected versus uninfected control animals, while the naïve (CD44^lo^ CD62L^hi^) cell compartment if anything was decreased in size (**Figure 1A**). As expected, viral Ag GP66-specific as well as NP309-specific memory CD4^+^ T cells were detected exclusively in the infected CD44^hi^ CD62L^lo^ cell population (**Figures 1B**, **Figure S1A**). These findings suggested that the CD44^hi^ CD62L^lo^ CD4^+^ T-cell population seen in infected animals represents a mixture of preexisting MP cells and LCMV-driven foreign Ag-specific memory cells.

To search for markers that are differently expressed in MP and foreign Ag-specific memory T lymphocytes, we compared the CD44^hi^ CD62L^lo^ cell population preexisting in the SPF environment with GP66-specific memory cells in infected mice by means of single-cell RNA sequencing (scRNAseq). Uniform Manifold Approximation and Projection (UMAP) analysis revealed that these two populations are transcriptomically distinct (**Figure 1C**), and subsequent scoring analysis indicated that expression of genes encoding positive regulators in the cell cycle (26) was higher in MP versus foreign Ag-specific memory cells, while the opposite was true for negative regulators (**Figure 1D**). Consistent with this finding, the uninfected CD44^hi^ CD62L^lo^ cell population displayed significantly higher Ki67 expression than did its counterpart in infected mice (**Figure 1E**), and closer analysis of the latter cell population revealed that GP66-specific memory cells are almost all Ki67^neg^ (**Figure 1F**). The above observations are in agreement with previous findings demonstrating that MP cells are rapidly proliferating while foreign Ag-specific memory cells are quiescent (22) and confirm the scRNAseq data shown in **Figure 1C**. Our data also support the hypothesis that the CD44^hi^ CD62L^lo^ CD4^+^ T-cell population is flexible in size, with CD44^hi^ CD62L^lo^ cells from infected mice, and especially their tetramer^neg^ subpopulation, representing a mixture of MP and foreign Ag-specific memory T lymphocytes since their Ki67^+^ fraction was intermediate in magnitude between those observed in uninfected CD44^hi^ CD62L^lo^ and infected foreign Ag-specific memory cells (as defined by GP66-tetramer staining) (**Figures 1E, F**).

Through further scoring analysis of the above scRNAseq data, we found that the expression of genes associated with memory T-cell formation (27) was significantly lower in the MP compared to the foreign Ag-specific memory compartment (**Figure 1D**). Because CD127 (IL-7 receptor α chain) and Sca1 (also referred to as Ly6a) are known to be expressed on foreign Ag-specific memory T lymphocytes (12, 28–30), we next compared their expression on CD4^+^ T cells in uninfected versus infected animals. MP (and minor population of CD44^hi^ CD62L^hi^) cells in uninfected mice were found to consist of CD127^hi^ Sca1^lo^, CD127^hi^ Sca1^hi^, CD127^lo^ Sca1^hi^, and CD127^lo^ Sca1^lo^ subsets, while naïve cells were largely CD127^hi^ Sca1^lo^ (**Figures 1G**, **S2A**). In CD44^hi^ CD62L^lo^ cells, the CD127^hi^ Sca1^hi^ but no other subpopulations increased in size in infected CD4^+^ T lymphocytes (**Figure 1G**). These results suggested that in comparison with heterogeneous MP cells, foreign Ag-specific memory cells are CD127^hi^ Sca1^hi^. Consistent with this hypothesis, GP66- as well as NP309-tetramer^+^ memory cells were essentially all CD127^hi^ Sca1^hi^, while the tetramer^neg^ cells that presumably represent a mixture of MP and foreign Ag-specific memory populations as described above contained a frequency of CD127^hi^ Sca1^hi^ cells intermediate between that in uninfected MP and tetramer^+^ memory cells (**Figures 1H**, **S1B**). Furthermore, in CD44^hi^ CD62L^lo^ CD4^+^ T lymphocytes, the CD127^hi^ Sca1^hi^ subset had the lowest Ki67 expression in both infected and uninfected mice (**Figures 1I, J**). Together, these data demonstrate that MP cells preexisting in an SPF environment are subdivided into four different populations based on CD127 and Sca1 expression, while foreign Ag-specific memory T lymphocytes are all CD127^hi^ Sca1^hi^, with both populations constituting the CD44^hi^ CD62L^lo^ CD4^+^ T-cell compartment at homeostasis.

### Self-Driven Memory-Phenotype T Lymphocytes Are Distinguishable From Foreign Antigen-Specific Memory Cells Based on Bcl2 Expression

**Figure 2:**
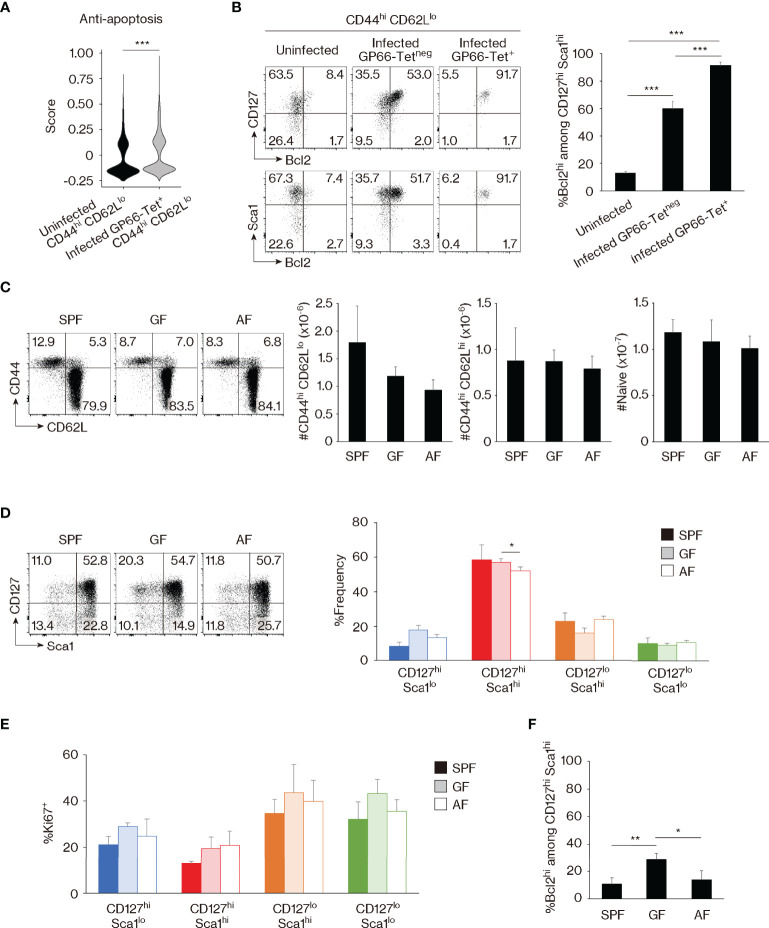
Bcl2 marks foreign antigen (Ag)-specific memory versus self-driven memory-phenotype (MP) cells. **(A)** Comparison of gene expression between MP versus foreign Ag-specific memory cells. The violin plot shows relative expression of anti-apoptotic genes across populations. The anti-apoptotic gene list is provided in *Materials and Methods* and **Table S1**. **(B)** Foreign Ag-specific memory cells are Bcl2^hi^. The representative dot plots display expression levels of CD127, Sca1, and Bcl2 in the indicated CD44^hi^ CD62L^lo^ cell populations, while the bar graph shows the frequency (mean ± SD) of Bcl2^hi^ cells among the indicated CD127^hi^ Sca1^hi^ subpopulations (n = 3–4 mice). Data are representative of 2 independent experiments. **(C, D)** Commensal or food Ags do not significantly contribute to generation of CD127^hi^ Sca1^hi^ MP cells in specific pathogen-free (SPF) environment. Dot plots depicting expression of **(C)** CD44 and CD62L in CD4^+^ T lymphocytes as well as **(D)** CD127 and Sca1 in MP cells from the indicated animals together with bar graphs indicating **(C)** the number (mean ± SD) of CD44^hi^ CD62L^lo^ (MP), CD44^hi^ CD62L^hi^, and CD44^lo^ CD62L^hi^ (naïve) CD4^+^ T cells as well as **(D)** the frequency (mean ± SD) of MP subpopulations among total MP cells from each group are displayed (n = 3–4 mice). Data are representative of 2 independent experiments performed. **(E, F)** Expression levels of Ki67 and Bcl2 in MP cells are largely unaltered in the absence of commensal and/or food Ags. **(E)** The bar graph shows the frequency (mean ± SD) of Ki67^+^ cells among the indicated MP subpopulations (n = 3–4 mice). **(F)** A bar graph indicating the frequency (mean ± SD) of Bcl2^hi^ cells among CD127^hi^ Sca1^hi^ MP cells from the indicated groups (n = 3–4 mice). Data are representative of 2 independent experiments. Statistically significant differences are indicated as *p < 0.05, **p < 0.01, and ***p < 0.001.

Because foreign Ag-driven memory cells are CD127^hi^ Sca1^hi^ and this phenotype is partially shared by MP cells (**Figures 1G, H**), we sought to determine whether or not MP lymphocytes with the same phenotype represent a subpopulation of foreign Ag-specific memory cells. For this purpose, we re-analyzed the scRNAseq data obtained in **Figure 1C** and found that expression of anti-apoptotic genes (31) was significantly lower in MP versus foreign Ag-specific memory cells (**Figure 2A**). Given that *Bcl2* plays a critical role as an anti-apoptotic factor in T lymphocytes (32), we measured its protein expression levels in these two populations. While CD127^hi^ Sca1^hi^ MP cells were largely Bcl2^lo^, ~90% of CD127^hi^ Sca1^hi^ GP66- and NP309-tetramer^+^ memory T lymphocytes were Bcl2^hi^ (**Figures 2B**, **S1C**). Thus, CD127^hi^ Sca1^hi^ MP cells appear to represent a unique population that is distinct from foreign Ag-specific memory T lymphocytes in terms of Bcl2 expression. In addition, we observed a small fraction (~10%) of Bcl2^hi^ cells in CD127^hi^ Sca1^hi^ MP CD4^+^ T cells, and this fraction was more enriched in CD44^hi^ CD62L^hi^ cells (**Figures 2B**, **S2B**). The significance of this finding will be discussed later.

To further investigate whether the development of the CD127^hi^ Sca1^hi^ MP subset is foreign Ag-dependent or Ag-independent, we examined MP cells from SPF, GF, and antigen-free (AF) mice, the latter being deprived of both food and commensal Ags (33). MP as well as CD44^hi^ CD62L^hi^ and naïve CD4^+^ T cells were essentially intact in SPF, GF, and AF mice (**Figure 2C**). Surprisingly, CD127^hi^ Sca1^hi^ MP cells were largely unchanged in the three animal groups (**Figure 2D**). In addition, Ki67 expression was not significantly increased in GF or AF mice, and Bcl2 levels were not decreased and instead were elevated in the former animals (**Figures 2E, F**). This was also the case in the minor CD44^hi^ CD62L^hi^ CD4^+^ T-cell population (**Figure S2C, D**). Thus, CD127^hi^ Sca1^hi^ Bcl2^lo^ MP cells can be generated in the absence of foreign Ags, presumably in response to self Ags.

### Among the Four Memory-Phenotype Subsets, CD127^hi^ Sca1^hi^ Cells Represent the Most Mature Subpopulation Generated From Peripheral Naïve Precursors

**Figure 3:**
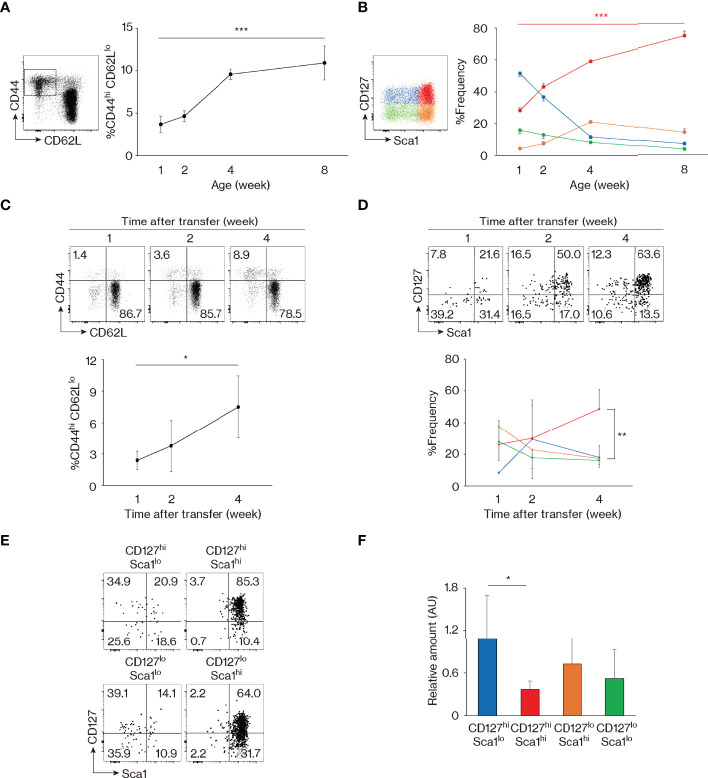
Memory-phenotype (MP) T lymphocytes are composed of four subsets representing different stages of maturation. **(A, B)** CD127^hi^ Sca1^hi^ MP cells develop with age. The graphs indicate the fractions (mean ± SD) of **(A)** CD44^hi^ CD62L^lo^ among CD4^+^ T cells and **(B)** each subpopulation among MP cells (n = 3–5 mice). Representative dot plots displaying expression of **(A)** CD44 and CD62L in CD4^+^ T lymphocytes and **(B)** CD127 and Sca1 in MP CD4^+^ T cells are also included. Data shown are pooled from 2 independent experiments performed. **(C, D)** CD127^hi^ Sca1^hi^ MP cells are generated from naïve precursors. Naïve CD4^+^ T lymphocytes sorted from CD45.2 mice were transferred to CD45.1 wild-type (WT) recipients and analyzed several weeks later. Dot plots show **(C)** CD44 and CD62L expression in the donor cell population and **(D)** CD127 and Sca1 levels in the newly generated MP donor cells, while the graphs indicate the frequency (mean ± SD) of **(C)** MP cells in the donor cell population and **(D)** the indicated subpopulations among MP donor cells (n = 3–4 mice). Data are representative of 2 independent experiments. **(E)** All four MP subsets eventually differentiate into CD127^hi^ Sca1^hi^ cells. Four MP subpopulations sorted from CD45.2 mice were transferred to CD45.1 WT recipients and analyzed for their CD127 and Sca1 expression 2 weeks later. Dot plots in the indicated donor MP subpopulations are shown. Data are representative of 4–5 recipient mice from 3 independent experiments performed. **(F)** CD127^hi^ Sca1^hi^ MP T lymphocytes have the lowest amount of TRECs. A bar graph indicating the amount (mean ± SD) of TRECs relative to *Gapdh* that was calculated by the ΔCt method in each MP subpopulation is depicted (n = 5 mice). Data are representative of 2 independent experiments performed. Statistically significant differences are indicated as *p < 0.05, **p < 0.01, and ***p < 0.001.

The above results identify CD127, Sca1, and Bcl2 as markers that are differently expressed in foreign Ag-specific memory versus MP cells and indicate that MP CD4^+^ T lymphocytes comprise 4 distinct subsets: CD127^hi^ Sca1^lo^, CD127^hi^ Sca1^hi^, CD127^lo^ Sca1^hi^, and CD127^lo^ Sca1^lo^. Because CD127^hi^ Sca1^hi^ MP cells are present in almost equal numbers in SPF, GF, and AF mice, self Ags are thought to be the major stimuli for the generation of CD127^hi^ Sca1^hi^ as well as the other MP subsets in an SPF environment. To address how the four MP subpopulations defined by these phenotypic markers are generated and maintained, we measured CD127 and Sca1 levels in MP cells from mice of different ages. As expected, MP cells were rare in 1-week-old animals, and most of these cells were CD127^hi^ Sca1^lo^ (**Figures 3A, B**). Thereafter, the proportion of CD127^hi^ Sca1^hi^ lymphocytes increased progressively with age, in parallel with the total MP pool size.

In the case of CD8^+^ MP cells, a subpopulation referred to as innate memory cells are directly generated in the thymus (34). To ask whether CD127^hi^ Sca1^hi^ CD4^+^ MP cells arise in the thymus, we prevented lymphocyte egress from the thymus by injecting mice with FTY720 for 2 weeks. As expected, numbers of both CD4^+^ and CD8^+^ single-positive thymocytes accumulated in the thymus (**Figure S3A**), and in parallel, naïve CD4^+^ T cells decreased in number in secondary lymphoid tissues (**Figures S3B, C**). By contrast, peripheral MP CD4^+^ T lymphocytes as well as their four subfractions were largely unaffected by the treatment (**Figures S3B–D**), suggesting that MP cells are self-maintained in the periphery once generated. Consistent with this conclusion, the four MP subpopulations were not altered by adult thymectomy (**Figure S3E**). Based on these results, it is unlikely that peripheral CD127^hi^ Sca1^hi^ and other MP subpopulations are actively replaced by emigrants generated in the thymus.

To test the possibility that CD127^hi^ Sca1^hi^ MP cells are generated from peripheral naïve precursors, we transferred sorted naïve CD4^+^ T lymphocytes into wild-type (WT) recipients and analyzed the donor cells 1 to 4 weeks later. MP cells developed slowly with time (**Figure 3C**), with the CD127^hi^ Sca1^hi^ subset dominating by 4 weeks after transfer (**Figure 3D**). Thus, the four MP subsets including the CD127^hi^ Sca1^hi^ fraction appear to be generated from naïve T lymphocytes in the periphery.

To further examine the dynamics of MP cell maintenance following their development, we sorted for CD127^hi^ Sca1^lo^, CD127^hi^ Sca1^hi^, CD127^lo^ Sca1^hi^, and CD127^lo^ Sca1^lo^ MP subpopulations and transferred them individually to WT recipients. When the donor cell population was analyzed 2 weeks later, Sca1^lo^ cells were found to increase their Sca1 expression, while Sca1^hi^ cells remained Sca1^hi^ (**Figure 3E**). In addition, some CD127^hi^ cells converted to CD127^lo^ and vice versa (**Figure 3E**). Overall, these data argue that while naïve cells are CD127^hi^ Sca1^lo^, as they differentiate into MP cells, they eventually acquire a CD127^hi^ Sca1^hi^ phenotype, either directly from CD127^hi^ Sca1^lo^ precursors or *via* CD127^lo^ intermediates. Consistent with this notion, when the levels of T-cell receptor (TCR) excision circles (TRECs) were measured in the four MP subpopulations sorted from intact mice, levels were the highest and lowest in CD127^hi^ Sca1^lo^ and CD127^hi^ Sca1^hi^ cell populations, respectively, with CD127^lo^ cells expressing TRECs at an intermediate level (**Figure 3F**). Together, these data identify CD127^hi^ Sca1^hi^ MP cells as the most mature of the four subsets, their generation reflecting extensive cell division of their precursors.

### Memory-Phenotype Subpopulations Are Maintained Through T-Cell Receptor and CD28 Signaling

We previously reported that, as a whole population, MP cells become less dependent on TCR signaling once generated (4). This notion was based on the observation that treatment of SPF mice with either anti-I-Aβ monoclonal antibody (mAb) or cyclosporin A significantly inhibits the generation of MP cells from naïve precursors but has only a negligible effect on their steady-state proliferation. While these data established a lesser dependence of MP cell maintenance on Ag recognition, it was still unclear whether this function is completely independent of TCR signaling.

**Figure 4:**
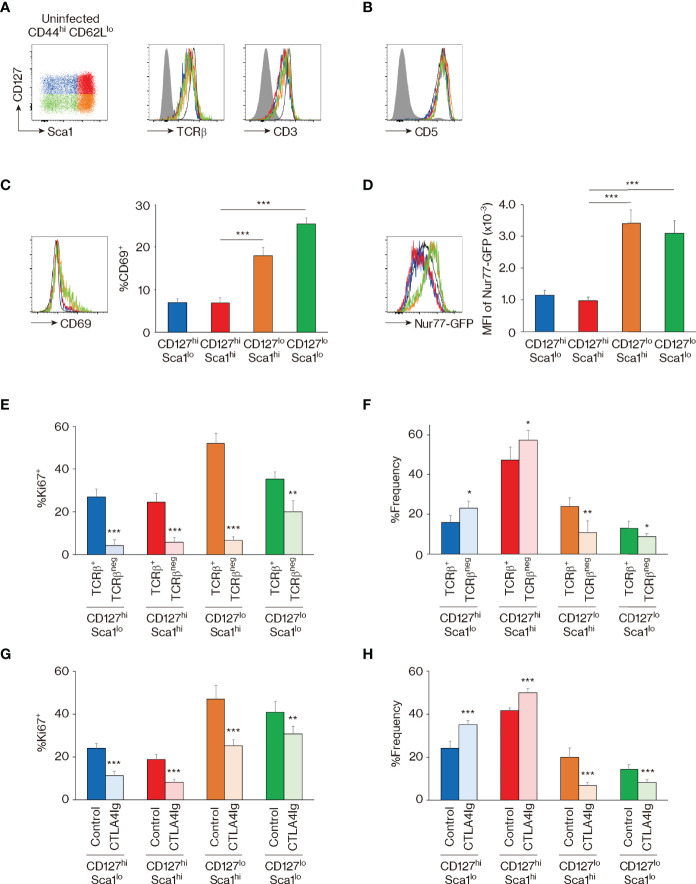
The four memory-phenotype (MP) subpopulations are maintained *via* T-cell receptor (TCR) and CD28 signaling. **(A–D)** CD127^lo^ MP subsets represent cell populations that have recently received TCR signaling. Representative histograms display **(A)** TCRβ, CD3, **(B)** CD5, **(C)** CD69, and **(D)** GFP expression in the indicated MP subpopulations from Nur77-GFP reporter mice, while the bar graphs indicate **(C)** the frequency (mean ± SD) of CD69^+^ and **(D)** the mean fluorescence intensity (MFI) (mean ± SD) of GFP among the MP subpopulations (n = 5 mice). Filled histograms show negative control staining, whereas black and open histograms display naïve CD4^+^ T cells. **(E, F)** TCR signaling is essential for proliferation and dynamic equilibrium of MP subpopulations. CD4-CreERT2 TCRα^flox^ mice received tamoxifen (TMX) and were analyzed 10 days later. Bar graphs indicating the frequency (mean ± SD) of **(E)** Ki67^+^ cells among both TCRβ^+^ and TCRβ^neg^ fractions from each MP subset and **(F)** the indicated subpopulations among the TCRβ^+^ or TCRβ^neg^ MP cells are depicted (n = 5 mice). **(G, H)** CD28 signals are critical for optimal maintenance of MP subpopulations. Mice received CTLA4-Ig and were analyzed 10 days later. The bar graphs show the frequency (mean ± SD) of **(G)** Ki67^+^ cells among the indicated MP subpopulations from each group and **(H)** the indicated subsets among total MP T lymphocytes (n = 5 mice). Data are representative of 2 independent experiments. Statistically significant differences are indicated as *p < 0.05, **p < 0.01, and ***p < 0.001.

To address this issue, we analyzed the strength of TCR signaling that MP subpopulations receive at baseline. Initial experiments revealed that the four MP subsets express equivalent levels of TCRβ and CD3 as well as CD5 (**Figures 4A, B**), a marker that reflects TCR affinity to self Ags (35). Nevertheless, in Nur77-GFP reporter mice where the sum of TCR signal strength that T cells receive is reflected by reporter expression (36), CD127^lo^ Sca1^hi^ and CD127^lo^ Sca1^lo^ MP subpopulations showed significantly higher levels of GFP as well as CD69 than did their CD127^hi^ counterparts (**Figures 4C, D**). Given that CD127^hi^ and CD127^lo^ subsets are interchangeable in the steady state (**Figure 3E**), it is possible that the former MP subpopulation downregulates CD127 immediately after TCR ligation.

To test this hypothesis *in vivo*, we utilized CD4-CreERT2 TCRα^flox^ mice in which we previously established that ~50% of MP cells lose their TCR expression as a consequence of tamoxifen (TMX) treatment (5). Thus, by examining the four MP subfractions in TCRβ^+^ and TCRβ^neg^ MP subpopulations 10 days after TMX treatment, we could compare their steady-state proliferation in the presence or absence of normal levels of tonic TCR signaling in the same mouse. Using these animals, we found that Ki67 expression was significantly reduced by TCR ablation in all four MP subpopulations (**Figure 4E**). Moreover, the CD127^hi^ fraction increased while the CD127^lo^ decreased in size when TCR levels were reduced (**Figure 4F**). These results show that the four MP subpopulations proliferate in the presence of tonic TCR signaling, presumably delivered by self Ag recognition, and further suggest the existence of TCR-dependent interchangeability between CD127^hi^ and CD127^lo^ MP cells in a dynamic steady state.

In addition to TCR engagement, CD28 ligation plays an essential role in MP cell maintenance (4). To assess the function of the same signaling pathway in the four MP subpopulations, we treated mice with CTLA4-Ig for 10 days. In a similar manner to that induced by TCR signal blockade, Ki67 expression was reduced in the CTLA4-Ig-treated group, and CD127^hi^ cells increased while their CD127^lo^ counterparts decreased in the same animals (**Figures 4G, H**). Thus, optimal proliferation and dynamic equilibrium of MP cell subpopulations require tonic CD28 engagement in addition to TCR signaling.

### CD127^hi^ Sca1^hi^ Memory-Phenotype Cells Have a Th1 Cytokine Signature

**Figure 5:**
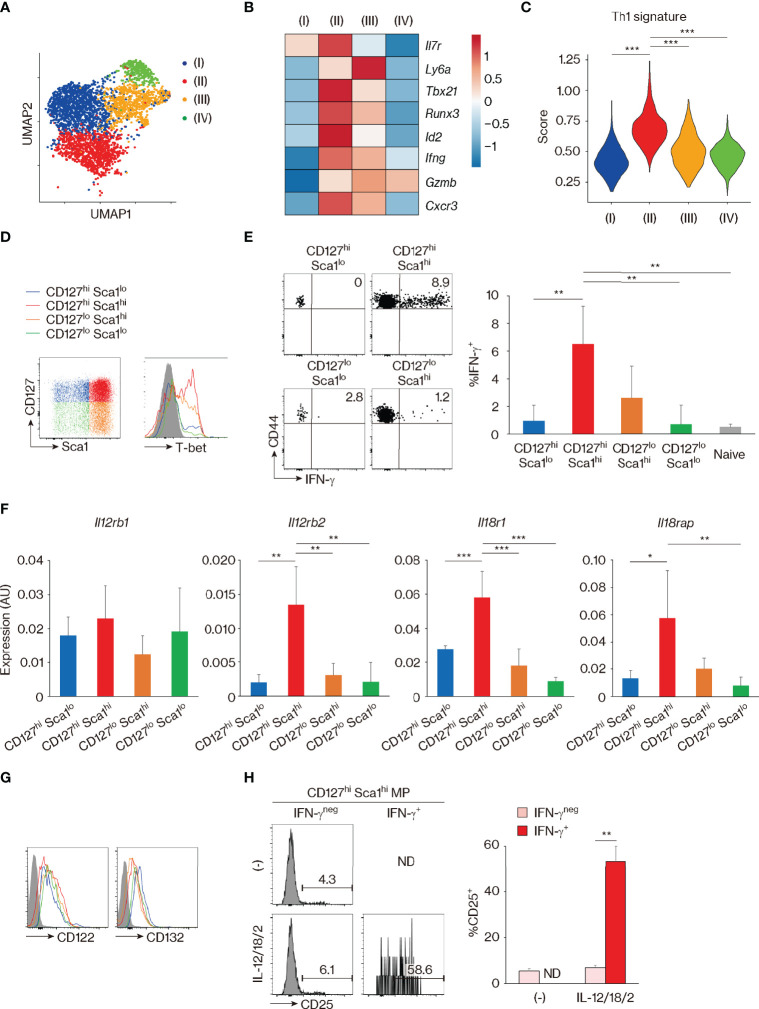
CD127^hi^ Sca1^hi^ memory-phenotype (MP) T lymphocytes can exert innate Th1-like activity *in vitro*. **(A–C)** Clustering analysis of MP T lymphocyte population. Single-cell RNA sequencing (scRNAseq) data of MP cells in Figure 1C were analyzed by unsupervised clustering. **(A)** The plot displays single cells determined by the Uniform Manifold Approximation and Projection (UMAP) algorithm. Each dot represents a cell, and colors highlight unsupervised cell clusters. Clusters representing less than 5% of the total MP population were excluded. **(B)** A heatmap showing row-standardized expression of selected genes among MP clusters. **(C)** A violin plot depicting relative expression of Th1-associated genes in the indicated MP clusters. **(D)** CD127^hi^ Sca1^hi^ MP cells express the highest levels of T-bet. A representative histogram showing T-bet levels in each MP subset from 3 mice is depicted. **(E)** CD127^hi^ Sca1^hi^ MP subpopulation can produce IFN-γ in response to Th1-differentiating cytokines in the absence of antigen (Ag) recognition. Sorted MP as well as naïve CD4^+^ T cell subpopulations were stimulated with IL-12, IL-18, and IL-2 for 24 h. The dot plots show IFN-γ production by the indicated cells, while the bar graph depicts the IFN-γ^+^ fraction (mean ± SD) among the MP and naïve subpopulations (n = 4 mice). Data shown are representative of 2 independent experiments performed. **(F)** The CD127^hi^ Sca1^hi^ MP subset expresses high amounts of *Il12rb2*, *Il18r1*, and *Il18rap*. Bar graphs showing relative expression levels of the indicated genes in each MP subpopulation are depicted (n = 4 mice). Data are representative of 2 independent experiments. **(G, H)** CD127^hi^ Sca1^hi^ MP cells express functional IL-2 receptors under the presence of Th1-differentiating cytokines. Representative histograms in **(G)** display CD122 and CD132 expression in each MP subset from 4 mice in steady state, while those in **(H)** show CD25 expression in IFN-γ^neg^ and IFN-γ^+^ fractions of sorted CD127^hi^ Sca1^hi^ MP cells that were cultured in the presence or absence of IL-12, IL-18, and IL-2 for 24 h. A bar graph indicating the frequency (mean ± SD) of CD25^+^ cells among each group is also depicted (n = 3 mice). Data are representative of 2 independent experiments performed. Statistically significant differences are indicated as *p < 0.05, **p < 0.01, and ***p < 0.001. ND, not detected.

The above observation that CD127, Sca1, and Bcl2 are differently expressed in MP and foreign Ag-driven memory CD4^+^ T cells revealed an unexpected phenotypic heterogeneity within the former lymphocyte population (**Figures 1**–**4**). This finding prompted us to ask whether these four MP cell subpopulations possess different functions. The experiments performed above defined MP subpopulations based on markers (CD127 and Sca1) that are well known to characterize foreign Ag-driven memory cells, but left open the possibility that other molecules might better characterize functional MP subsets. To address this question, we analyzed the MP cell population in the scRNAseq dataset generated in **Figure 1C** by unsupervised clustering, to define its components based on the full transcriptome rather than on predefined markers. This analysis divided the MP cell population into four clusters (I–IV) (**Figures 5A, B**). Comparing the expression of *Il7r* and *Ly6a* among these four clusters showed a clear demarcation into *Il7r* ^hi^ *Ly6a* ^lo^, *Il7r* ^hi^ *Ly6a* ^hi^, *Il7r* ^lo^ *Ly6a* ^hi^, and *Il7r* ^lo^ *Ly6a* ^lo^ gene expression patterns for clusters (I), (II), (III), and (IV), respectively; importantly, these correspond respectively to the CD127^hi^ Sca1^lo^, CD127^hi^ Sca1^hi^, CD127^lo^ Sca1^hi^, and CD127^lo^ Sca1^lo^ MP subpopulations defined in **Figure 1G**. The results of this analysis thus provide independent support to the conclusion that CD127 and Sca1 expression define four transcriptomically distinct MP subpopulations. Further clustering and scoring analyses revealed that the cluster (II), that is *Il7r* ^hi^ *Ly6a* ^hi^ and thus equivalent to the CD127^hi^ Sca1^hi^ MP subpopulation, has the highest Th1-associated gene signature (27) (**Figures 5B, C**), suggesting that this subset displays Th1-related function.

We have previously shown that MP CD4^+^ T lymphocytes can respond to IL-12 and produce IFN-γ *in vivo* (4). Consistent with this finding, when whole splenocytes were cultured in the presence of IL-12, IL-18, IL-2, or a combination of these cytokines *in vitro*, CD4^+^ MP cells produced IFN-γ in response to IL-12 (**Figure S4A**). The latter response increased with the addition of IL-18 and IL-2 and peaked at 24–48 h after cytokine stimulation (**Figures S4A, B**). With this *in vitro* system, we asked which of the MP subpopulation(s) can generate the above-described Th1-like response. To do so, we first checked the expression of T-bet, which we previously reported to play an essential role in the determination of Th1-like MP cell activity (4, 5). As shown in **Figure 5D**, CD127^hi^ Sca1^hi^ cells expressed the highest levels of T-bet, confirming the result of clustering analysis in **Figure 5B**. Consistent with this observation, when each MP subpopulation as well as naïve cells was sort-purified and cultured individually in the presence of IL-12, IL-18, and IL-2, CD127^hi^ Sca1^hi^ MP cells produced high amounts of IFN-γ (**Figure 5E**). Furthermore, the same MP subpopulation expressed high levels of *Il12rb2*, *Il18r1*, and *Il18rap* in the steady state (**Figure 5F**). In terms of IL-2 receptor expression, CD122 and CD132 were expressed on all four MP subsets (**Figure 5G**). However, CD25 (IL-2 receptor α chain) was only induced when CD127^hi^ Sca1^hi^ MP cells were stimulated by Th1 cytokines (**Figure 5H**), consistent with previous reports showing that IL-12 upregulates CD25 on Th1 cells (37, 38). Together, these results identify CD127^hi^ Sca1^hi^ MP T lymphocytes as a resting population with selective Th1 effector potential.

**Figure 6:**
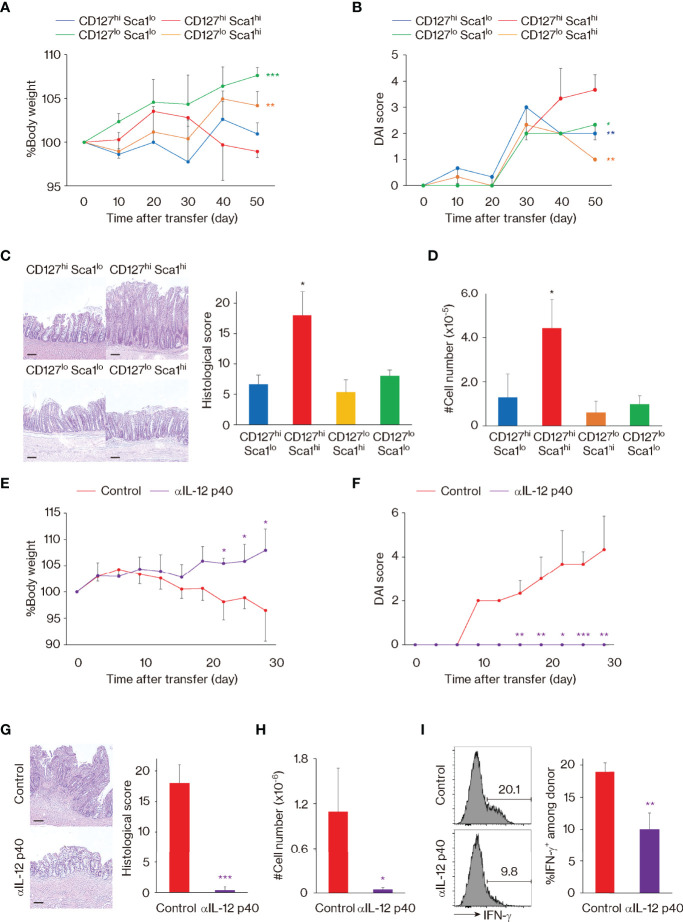
The CD127^hi^ Sca1^hi^ memory-phenotype (MP) cell subset can induce colitis in *Rag2* knockout (KO) mice. **(A–D)** Among the four MP subsets, CD127^hi^ Sca1^hi^ cells can induce the most severe colitis. Sorted MP subpopulations were individually transferred to *Rag2* KO recipient mice. Graphs indicate **(A)** relative body weight (mean ± SD) and **(B)** disease activity index (DAI) score (mean ± SD) in each group at different time points as well as **(C)** histological score (mean ± SD) and **(D)** the number (mean ± SD) of donor cells in the colon on day 50 (n = 3 mice). Representative images showing histology of the colon from each group are also included in panel **(C)**. **(E–I)** Blockade of IL-12 p40 ameliorates MP-induced colitis. Sorted CD127^hi^ Sca1^hi^ MP cells were transferred to *Rag2* KO mice that were then treated with anti-IL-12 p40 mAb or control IgG. Graphs show **(E)** relative body weight (mean ± SD) and **(F)** DAI score (mean ± SD) in each group at different time points as well as **(G)** histological score (mean ± SD), **(H)** the number (mean ± SD) of donor cells, and **(I)** their IFN-γ^+^ fraction (mean ± SD) in the colon on day 28 (n = 3 mice). Histological images of the colon from each group on day 28 are displayed in **(G)**. Data shown are representative of 2 independent experiments. Scale bars in histological images show 100 μm. Statistically significant differences are indicated as *p < 0.05, **p < 0.01, and ***p < 0.001.

Finally, we sought to validate the above functional observations *in vivo*. Naïve CD4^+^ T lymphocytes are known to induce severe colitis when transferred to *Rag2* knockout (KO) mice (39, 40). Using this approach, we tested the capacity of MP cell subpopulations to induce colitis by individually transferring CD127^hi^ Sca1^lo^, CD127^hi^ Sca1^hi^, CD127^lo^ Sca1^hi^, and CD127^lo^ Sca1^lo^ Foxp3^−^ MP CD4^+^ T cell subsets from Foxp3-reporter mice to *Rag2* KO recipients. CD127^hi^ Sca1^hi^ cells were found to induce mild body weight loss and clinical as well as histological colitis (**Figures 6A–C**). Consistent with this observation, the total number of recovered donor cells was the highest in the CD127^hi^ Sca1^hi^ recipient group (**Figure 6D**). Thus, among the four MP subpopulations, the CD127^hi^ Sca1^hi^ subset induces the most severe colitis when transferred to *Rag2* KO mice. We further asked whether colitis driven by the CD127^hi^ Sca1^hi^ MP subpopulation is dependent on IL-12 and/or IL-23. To do so, we blocked IL-12 p40 using mAb specific for the cytokine after donor cell transfer. As shown in **Figures 6E–G**, body weight loss, clinical symptoms, and histological colitis were almost completely abrogated as a result of mAb treatment. Consistent with this, the total number of donor cells and their IFN-γ^+^ fraction were dramatically reduced in the same mAb-treated animals (**Figures 6H, I**). These data suggest that in *Rag2* KO mice, CD127^hi^ Sca1^hi^ MP cells can generate inflammatory responses, thus supporting their functional relevance.

## Discussion

CD4^+^ T lymphocytes with a memory phenotype present in normal, unimmunized animals have been known for more than 30 years and have been presumed to comprise a mixture of explicit foreign Ag-specific “authentic” memory and foreign Ag-independent MP cells (1–3). A number of previous studies have addressed the features of these two CD4^+^ T-cell populations. Thus, we and other groups showed that nearly all conventional foreign Ag-specific memory cells are resting cells with a slow turnover and are largely major histocompatibility complex (MHC)-independent while heavily dependent on the cytokines IL-7 and/or IL-15. In contrast, naturally arising MP cells comprise a mixture of fast-proliferating, MHC-dependent cells together with slowly proliferating, IL-7-dependent cells (12, 22, 23). However, a definitive comparison of these subsets was not possible because of the lack of phenotypic markers.

Our present study addressed this long-standing problem by searching for phenotypic differences between MP and foreign Ag-specific memory CD4^+^ T cells. As shown here, we have identified CD127, Sca1, and Bcl2 as key markers that are differently expressed in these two lymphocyte populations. Thus, our data demonstrate that foreign Ag-specific memory T lymphocytes comprise a homogeneous population of CD127^hi^ Sca1^hi^ Bcl2^hi^ cells, while MP cells are a heterogeneous mixture of CD127^lo~hi^ Sca1^lo~hi^ that are mostly Bcl2^lo^ (summarized in **Figure S5**). While in the case of CD8^+^ T lymphocytes T_VM_ and foreign Ag-specific memory cells have been previously reported to be distinguishable from each other based on their levels of integrins α4, α1, and β1 as well as NKG2D (41, 42), our findings are the first to demonstrate differential marker expression between CD4^+^ MP and foreign Ag-specific memory cells and strongly support the notion that these two cell types are qualitatively distinct. Furthermore, employing these markers, we have identified distinct types of subsets within the MP population, with inflammatory activity predominantly associated with the CD127^hi^ Sca1^hi^ phenotype.

The mechanisms responsible for the generation and maintenance of foreign Ag-specific memory CD4^+^ T lymphocytes are well defined (2). Thus, in conventional adaptive immune responses against pathogens, naïve T lymphocytes that are specific for challenge Ags robustly proliferate to give rise to effector cells that contribute to host defense. After pathogen clearance, most effector cells die, leaving a small residual population of foreign Ag-specific memory cells that protects the host from secondary infection with the same pathogen. For memory cells to exert such long-term host-protective function, they need to survive long term in the absence of stimulation by cognate foreign Ags. IL-7 serves a major role in this function (12, 14, 15). This cytokine triggers T cells through its receptor to promote their survival by inducing Bcl2 upregulation and basal turnover in memory cells. In this context, our finding that foreign Ag-specific memory cells express high levels of CD127 and Bcl2 is not surprising.

In contrast, the mechanisms responsible for MP cell maintenance have been less clearly understood (1–3). In the past, this process has been investigated primarily by transferring MP cells to congenic, usually lymphopenic, recipient mice and analyzing the donor cells 1~2 weeks later. Previous reports established that under such situations, MP CD4^+^ T lymphocytes exhibit two different types of cell division (i.e., slow and fast homeostatic proliferation) as compared to foreign Ag-specific memory cells that exhibit mild and homogeneous cell expansion, and that slow proliferation requires IL-7 while rapid cell division is more dependent on TCR and costimulatory signaling (11–13). Here we provide new evidence suggesting that IL-7-dependent slow expansion can occur homeostatically in physiologic, non-transferred conditions since we observed that some MP cells express functional IL-7 receptors as evidenced by the joint presence of CD127 and CD132 (**Figures 1G**, **5G**) and that such CD127^hi^ subsets display lower levels of Ki67 than their CD127^lo^ counterparts (**Figure 1J**). In addition, under these normal lymphoreplete conditions, TCR-dependent fast cell division was the most conspicuous for CD127^lo^ MP cells and was marked by a Ki67^+^ phenotype (**Figure 1J**) together with evidence of strong TCR and CD28 signaling (**Figure 4**).

In lymphosufficient conditions, we previously suggested that rapid proliferation of MP CD4^+^ T lymphocytes is balanced by comparable cell death (4), a situation distinct from that observed with quiescent foreign Ag-specific memory cells (15, 22). Our present data are consistent with this difference since Bcl2, which is critical for T lymphocyte longevity (32), was significantly lower in MP as compared to foreign Ag-specific memory cells (**Figure 2B**). This low Bcl2 expression in MP cells was maintained after LCMV infection, a situation that is particularly apparent in the CD127^lo^ or Sca1^lo^ subsets within the CD44^hi^ CD62L^lo^ population in infected mice (**Figure 2B**). Together with our observation that the CD127^lo^ or Sca1^lo^ subsets (i.e., CD127^hi^ Sca1^lo^, CD127^lo^ Sca1^hi^, and CD127^lo^ Sca1^lo^ cells) were largely unchanged after LCMV infection (**Figure 1G**), these data argue that the Bcl2^lo^ as well as CD127^lo~hi^ and Sca1^lo~hi^ phenotype is a unique feature of MP T lymphocytes regardless of whether animals have been pathogen exposed. The above findings are also consistent with our hypothesis based on previous studies with *Toxoplasma* infection that during the early stages following pathogen challenge, the short-lived Bcl2^lo^ MP cell population contributes to transient host protection until this function is replaced through the development of Bcl2^hi^ foreign Ag-specific memory cells (4, 5).

In addition to the large low Bcl2 expression in MP cells, we observed that ~10% of CD127^hi^ Sca1^hi^ MP cells are Bcl2^hi^ (**Figures 2B**, **S1C**). In this regard, a previous study pointed out that MP cells contain a minor fraction of cells that closely resemble Ag-specific memory cells, although whether the Ags driving the MP cells are foreign or self was unclear (12). In the present study, the minor CD127^hi^ Sca1^hi^ Bcl2^hi^ MP cells were not reduced in GF or AF mice and even increased in the former animals (**Figure 2F**), arguing that these cells can arise through contact with self Ags, although the explanation for why the same fraction was elevated in GF mice remains unclear. Interestingly, the Bcl2^hi^ MP cell compartment was enriched in CD44^hi^ CD62L^hi^ CD4^+^ T cells (**Figure S2**), i.e., the counterpart of the “central memory” cells that form the bulk of CD44^hi^ MP CD8^+^ T lymphocytes (41, 43). These CD62L^hi^ cells may be viewed as fully differentiated MP cells, for both CD4^+^ and CD8^+^ T cells.

For CD4^+^ MP cells, the relative paucity of CD44^hi^ CD62L^hi^ cells correlates with low expression of CD122 on CD4^+^ versus CD8^+^ T cells, thereby making mature CD4^+^ MP cells poorly reactive to IL-15 and therefore largely dependent on IL-7 for their survival (12). Since levels of IL-7 are low in lymphoreplete animals (44), one can envision that survival of MP CD4^+^ T cells is heavily dependent on contact with self Ag/MHC complexes, with only a few of the responding cells able to slowly differentiate into an MHC-independent resting state. This small fraction of Bcl2^hi^ cells may reflect the self-driven counterparts of foreign Ag-specific memory CD4^+^ T cells. In the case of the latter population, their rapid switch to a fully differentiated state after initial induction may simply reflect that, unlike MP cells, foreign Ag-specific memory cells rapidly lose contact with stimulatory Ags when the pathogen concerned is eliminated, forcing the surviving cells to become MHC-independent.

The factors controlling the generation and maintenance of MP cells through contact with available ligands including self Ags are still poorly understood (1–3). For CD4^+^ MP cells, it is striking that the component of fast-proliferating cells in lymphoreplete hosts is far more conspicuous than for CD8^+^ cells (12, 16) and, as mentioned above, is accompanied by prominent signs of TCR activation on the responding CD4^+^ T cells (**Figures 4C, D**). The reason for this difference is unclear but it could reflect higher intrinsic TCR affinity for self ligands by CD4^+^ than CD8^+^ T cells. Thus, CD5 expression is substantially higher on naïve CD4^+^ than CD8^+^ T cells (45). Furthermore, this higher affinity to self Ags in CD4^+^ T lymphocytes may account for their capacity to regulate T-cell homeostasis. Indeed, a previous report showed that CD4^+^ T cells that react to subthreshold, endogenous peptide ligands compete with other T-cell clones with similar TCR specificities, thus preventing their outgrowth (46). However, why some naïve T lymphocytes spontaneously initiate responses to self Ags and then differentiate into resting cells remains unclear.

Despite their still undefined origin, MP CD4^+^ T cells can exert host protective effector function in response to IL-12 (4, 5). Our finding that these cells vary in their expression of CD127, Sca1, and Bcl2 allowed us to examine which subsets of CD4^+^ MP cells possess this cytokine reactivity. We observed that the capacity to respond to IL-12 and exert Th1-dependent inflammation *in vitro* is controlled largely by the mature CD127^hi^ Sca1^hi^ subset (**Figure 5**). We further identified that the same subset induces the most severe colitis when transferred to *Rag2* KO mice (**Figure 6**), demonstrating its potential role in inflammation *in vivo*. Because in the present study we employed an artificial transfer model where MP subsets are individually transferred to host mice deficient in B and T lymphocytes including regulatory T cells (Tregs), further investigation using more physiological settings is needed to confirm this proposed function.

The above evidence that CD127^hi^ Sca1^hi^ MP cells can exhibit inflammatogenic properties *in vitro* and *in vivo* supports our previous hypothesis that MP cells may have the ability to drive and/or exacerbate inflammation because of their self-specificity and tonic T-bet expression (4, 5). Given that MP cells can also contribute to Th1 cytokine-dependent host resistance to infection (4, 5), the inflammatory CD127^hi^ Sca1^hi^ subset can be regarded as a double-edged sword. This raises the interesting question of how their potentially immunopathologic function is inhibited in healthy animals. One possibility is that the activity of MP T lymphocytes is normally dampened by Tregs. In support of this notion, acute depletion of Tregs is known to induce systemic inflammation even in the absence of foreign Ags (47, 48). In addition, pathological autoreactivity might be repressed by other cell-intrinsic mechanisms. Indeed in CD8^+^ T cells, TCR signaling is known to be inhibited by upregulation of CD5 and CD45 and by downregulation of CD8 (49–51). Whether this is also the case in CD4^+^ MP lymphocytes remains to be investigated.

The findings reported here define MP CD4^+^ T lymphocytes as a unique but heterogeneous population that differs from conventional foreign Ag-specific memory cells in marker expression and establishes functional relevance for the mature subset of CD127^hi^ Sca1^hi^ MP cells. With defined markers now available, it will be of interest to search for further phenotypic and functional differences between the two cell types in future studies. Similarly, it will be important to determine if similar phenotypic differences between self-driven MP and foreign Ag-specific memory cells occur in humans. The existence of an MP-like population in humans is suggested by the observation of CD4^+^ T lymphocytes with an activated phenotype in cord blood and fetal tissues where an encounter with foreign Ags is likely to be very limited or non-existent (52, 53). Should such a population exists, one would predict it to display a low-level expression of CD127 and Bcl2. If an MP subpopulation can be demonstrated in humans, the subset could serve as a potential therapeutic target, either by boosting its activity to limit infection or by inhibiting its function to treat autoimmune and other inflammatory conditions.

## Materials and Methods

### Mice

C57BL/6 CD45.2^+^ WT mice were purchased from Taconic Biosciences (Rensselaer, NY, USA) or Japan SLC (Hamamatsu, Japan). *Rag2* KO and CD45.1^+^ WT mice were obtained from the National Institute of Allergy and Infectious Diseases (NIAID) contract facility at Taconic Biosciences or breeding stock maintained at Tohoku University Graduate School of Medicine. Nur77-GFP and Foxp3-RFP reporter mice were obtained from Jackson Laboratory (Bar Harbor, ME, USA). CD4-CreERT2 TCRα^flox^ mice are previously described (5). All mice were maintained in SPF animal facilities in the NIAID, National Cancer Institute (NCI), National Institutes of Health (NIH), or Tohoku University Graduate School of Medicine except for GF and AF mice, which were bred and maintained in the animal facility of Pohang University of Science and Technology as previously described (33). All mice were used at the age of 8–16 weeks except in the case of **Figures 3A, B**, where mice of indicated ages were utilized. The care and handling of the animals used in our studies were in accordance with the animal study protocols approved by the NIAID or NCI Animal Care and Use Committee, by the Institutional Committee for the Use and Care of Laboratory Animals of Tohoku University, or by the Institutional Animal Care and Use Committees of the Pohang University of Science and Technology.

### Thymectomy

Thymectomy was performed on 8~12-week-old mice under aseptic conditions. Sham thymectomy was conducted with the same procedure except that thymic lobes were left intact. At the end of the experiments, the thymic deficiency was confirmed by careful macroscopic inspection.

### Lymphocytic Choriomeningitis Virus Infection

LCMV Armstrong was propagated in baby hamster kidney-21 fibroblast cells [American Type Culture Collection (ATCC)]. Viral titers were determined by plaque assay using Vero African-green-monkey kidney cells (ATCC). Viral stocks were frozen at −80°C until use. For infection, mice were intraperitoneally injected with 2 × 10^5^ plaque-forming unit/mouse of the virus as previously described (22).

### *In Vivo* Chemical and mAb Treatment

To activate CreERT2 recombinases, mice received an intraperitoneal injection of TMX (20 mg/mouse) dissolved in corn oil (both Sigma-Aldrich, St. Louis, MO, USA) as previously described (5). To block IL-12 p40 or CD80/86 signals, anti-IL-12B p40 (C17.8), CTLA4-Ig, or control IgG (300 μg/20 g body weight; all from Bio X Cell, West Lebanon, NH, USA) were administered every 3 days as reported (4, 5). To inhibit lymphocyte egress from the thymus and lymph nodes, FTY720 (20 μg/20 g body weight; Cayman Chemicals, Ann Arbor, MI, USA) dissolved in phosphate-buffered saline (PBS) or control PBS was given to mice every day (20).

### Cell Sorting and Adoptive Transfer

Total CD4^+^ T lymphocytes were obtained from pooled splenocytes and lymph node cells of donor mice using a CD4^+^ T Cell Isolation Kit or CD4 Microbeads (Miltenyi Biotec, Bergisch Gladbach, Germany). Naïve CD4^+^ T cells were then purified by sorting for CD4^+^ CD25^neg^ CD44^lo^ CD62L^hi^ cells using a fluorescence-activated cell sorting (FACS) Aria II (BD Biosciences, San Jose, CA, USA). To obtain MP cell subsets, CD127^hi^ Sca1^lo^, CD127^hi^ Sca1^hi^, CD127^lo^ Sca1^hi^, and CD127^lo^ Sca1^lo^ subpopulations among CD4^+^ CD25^neg^ CD44^hi^ CD62L^lo^ cells from WT mice or CD4^+^ Foxp3^neg^ CD44^hi^ CD62L^lo^ cells from Foxp3-RFP reporter mice were sorted. Purity was >96%. For adoptive transfer experiments, depending on the experiments, 2 × 10^5^ to 1 × 10^6^ donor cells were intravenously injected into recipient animals.

### Assessment of Severity of Colitis

After MP subpopulations were transferred to *Rag2* KO mice, the animals were monitored for body weight. The disease activity index (DAI) score was assessed based on clinical symptoms as previously described (54). The histological score of the proximal portion of the colon was measured as previously reported (55).

### Flow Cytometric Analysis

Single-cell suspensions were prepared from spleens and red blood cells lysed in ACK buffer. In some experiments, splenic cells were further enriched for CD4^+^ T lymphocytes using a CD4^+^ T Cell Isolation Kit or CD4 Microbeads (Miltenyi Biotec). To obtain colonic cells, lamina propria mononuclear cells were isolated as previously described (54). Cells were suspended in staining buffer (PBS supplemented with 2% fetal bovine serum (FBS)) and incubated with CD16/32 mAb (2.4G2; Harlan Bioproducts, Indianapolis, IN, USA) for 10 min on ice. Cells were then incubated with the following mAbs or their combination for 20 min on ice: CD4 (RM4-5), CD8 (53-6.7), CD44 (IM7), CD62L (MEL-14), anti-NK1.1 (PK136), anti-TCRβ (H57-597) (Thermo Fisher Scientific, Waltham, MA, USA), CD3 (17A2), CD5 (53-7.3), CD25 (PC61), CD45.1 (A20), CD45.2 (104), CD69 (H1.2F3), CD122 (TM-β1), CD127 (A7R34), CD132 (TUGm2) (BioLegend, San Diego, CA, USA), and Sca1 (D7) (BD Biosciences). Tetramer staining used CD1d (PBS-57), I-Aβ-GP66, and I-Aβ-NP309 tetramers, obtained from the NIH Tetramer Core Facility (Emory University, Atlanta, GA, USA). To detect intracellular products, cells were fixed and permeabilized using Foxp3/Transcription Factor Staining Buffer Set for 30 min on ice after surface staining and then stained with mAbs against Bcl2 (10C4), Foxp3 (FJK-16s), Ki67 (SolA15) (Thermo Fisher Scientific), and/or IFN-γ (XMG1.2) (BioLegend) for 20 min on ice. For T-bet detection, fixed cells were stained with anti-T-bet (O4-46; BD Biosciences) mAb for 2 h at room temperature. Flow cytometry was performed using either Canto II, LSR II, Fortessa, or Symphony cytometers, and the data were analyzed with FlowJo software (BD Biosciences). Gating strategies are previously described (4, 5) and briefly summarized in **Figure S6**.

### Single-Cell RNA Sequencing Analysis

MP and foreign Ag-specific memory CD4^+^ T lymphocytes were obtained by sorting for TCRβ^+^ CD4^+^ CD25^neg^ CD44^hi^ CD62L^lo^ CD1d-tetramer^neg^ cells from uninfected mice and TCRβ^+^ CD4^+^ CD25^neg^ CD44^hi^ CD62L^lo^ GP66-tetramer^+^ cells from LCMV-infected mice, respectively. Sorted cells were then loaded onto the 10× Chromium platform using the Chromium Single Cell 3′ Library & Gel Bead Kit V2 according to the manufacturer’s instructions (56). Libraries were sequenced on the Illumina NextSeq using paired-end 26 × 98 bp, and sequencing files were processed to extract count matrices using the Cell Ranger Single Cell Software Suite (v2.2.0). Further analyses were performed in R using the Seurat package (3.0) (57). Scoring analyses for memory and Th1 signatures were performed as previously described (27). For cell cycle and anti-apoptotic signatures, genes were determined based on previous studies (26, 31) (**Table S1**). The obtained dataset is deposited on the Gene Expression Omnibus (GEO) (GEO number: GSE145999; token: kdspeciofrwhdod).

### Real-Time qPCR

For detection of *Il12rb1*, *Il12rb2*, *Il18r1*, and *Il18rap* mRNA, total RNA was extracted from sorted cells using the RNeasy Mini Kit (Qiagen, Hilden, Germany) and reverse-transcribed with SuperScript III Reverse Transcriptase (Thermo Fisher Scientific). For detection of TRECs, total DNA was isolated from sorted cells using the DNeasy Blood & Tissue Kit (Qiagen). Real-time PCR was performed using the THUNDERBIRD SYBR qPCR Mix (Toyobo, Osaka, Japan). qPCR analysis was carried out using a 7500 real-time PCR system (Thermo Fisher Scientific). Relative gene expression was calculated by the ΔCt method and normalized to the amount of *Gapdh*. The following primer sets were used: *Il12rb1*, 5′-CCCCAGCGCTTTAGCTTT-3′ and 5′-GCCAATGTATCCGAGACTGC-3′ (58); *Il12rb2*, 5′-AATTCTTCTTCACTTCCGCATACG-3′ and 5′-GCTCCCAGAAGCATTTAGAAAGT-3′ (59); *Il18r1*, 5′-GCTCAGACCCTAATGTGCAAG-3′ and 5′-TGCAGTTTGCCTTCAGAAATC-3′; *Il18rap*, 5′-TGCAATGAAGCGGCATCTGT-3′ and 5′-CCGGTGATTCTGTTCAGGCT-3′ (60); TRECs, 5′-CATTGCCTTTGAACCAAGCTG-3′ and 5′-TTATGCACAGGGTGCAGGTG-3′ (61); and *Gapdh*, 5′-CCAGGTTGTCTCCTGCGACTT-3′ and 5′-CCTGTTGCTGTAGCCGTATTCA-3′ (4).

### *In Vitro* Cell Culture and Cytokine Detection

In **Figures 5**, **S4**, total splenocytes or FACS-sorted MP subsets were stimulated with IL-12p70 (10 ng/ml; PeproTech, Cranbury, NJ, USA), IL-18 (10 ng/ml; MBL, Nagoya, Japan), and/or IL-2 (10 ng/ml; Ajinomoto, Tokyo, Japan) in Roswell Park Memorial Institute (RPMI) complete media for the indicated period at 37°C. At the last 6 h of incubation, Brefeldin A (1 μg/ml; BioLegend) was added. In **Figure 6I**, total cell suspensions were incubated in RPMI complete media supplemented with phorbol myristate acetate (PMA) (20 ng/ml) and ionomycin (1 μg/ml; both from Sigma-Aldrich) for 5 h at 37°C in the presence of Brefeldin A. Cells were then harvested and subjected to intracellular staining as described above.

### Statistical Analysis

A Student’s *t*-test was employed to establish statistical significance. *p*-Values <0.05 were considered significant.

## Data Availability Statement

The datasets presented in this study can be found in online repositories. The names of the repository/repositories and accession number(s) can be found below: https://www.ncbi.nlm.nih.gov/geo/, GSE145999.

## Ethics Statement

The animal study was reviewed and approved by the NIAID/NCI Animal Care and Use Committee, the Institutional Committee for the Use and Care of Laboratory Animals of Tohoku University, or the the Institutional Animal Care and Use Committees of the Pohang University of Science and Technology.

## Author Contributions

TK and AS designed the research. TK, TC, KK, ST, AK, TS, and RT performed the experiments. NI, DJ, JZ, JS, and RB provided collaborative support. TK, JS, and AS wrote the manuscript. All authors listed have made a substantial, direct, and intellectual contribution to the work and approved it for publication.

## Funding

This work was supported in part by the Intramural Research Programs of the NIAID and the NCI, Center for Cancer Research, NIH. TK also received support from the Japan Society for the Promotion of Science, Astellas Foundation for Research on Metabolic Disorders, Daiichi Sankyo Foundation of Life Science, Kobayashi Foundation, Mochida Memorial Foundation for Medical and Pharmaceutical Research, Life Science Foundation of Japan, Ohyama Health Foundation, Senshin Medical Research Foundation, Takeda Science Foundation, The Cell Science Research Foundation, The Chemo-Sero-Therapeutic Research Institute, The Mitsubishi Foundation, The Sumitomo Foundation, The Uehara Memorial Foundation, and The Waksman Foundation of Japan. In addition, TK received funding from Bristol-Myers Squibb. The funder was not involved in the study design, collection, analysis, interpretation of data, the writing of this article or the decision to submit it for publication.

## Conflict of Interest

The authors declare that the research was conducted in the absence of any commercial or financial relationships that could be construed as a potential conflict of interest.

## Publisher’s Note

All claims expressed in this article are solely those of the authors and do not necessarily represent those of their affiliated organizations, or those of the publisher, the editors and the reviewers. Any product that may be evaluated in this article, or claim that may be made by its manufacturer, is not guaranteed or endorsed by the publisher.
