# Supplementary material for: Redefining the Foreign Antigen and Self-Driven Memory CD4+ T-Cell Compartments via Transcriptomic, Phenotypic, and Functional Analyses
Source: Front Immunol. 2022 May 30;13:870542. doi: 10.3389/fimmu.2022.870542 (PMC9190281; doi:10.3389/fimmu.2022.870542)
Supplement: Supplementary file 1 [file DataSheet_1.pdf]

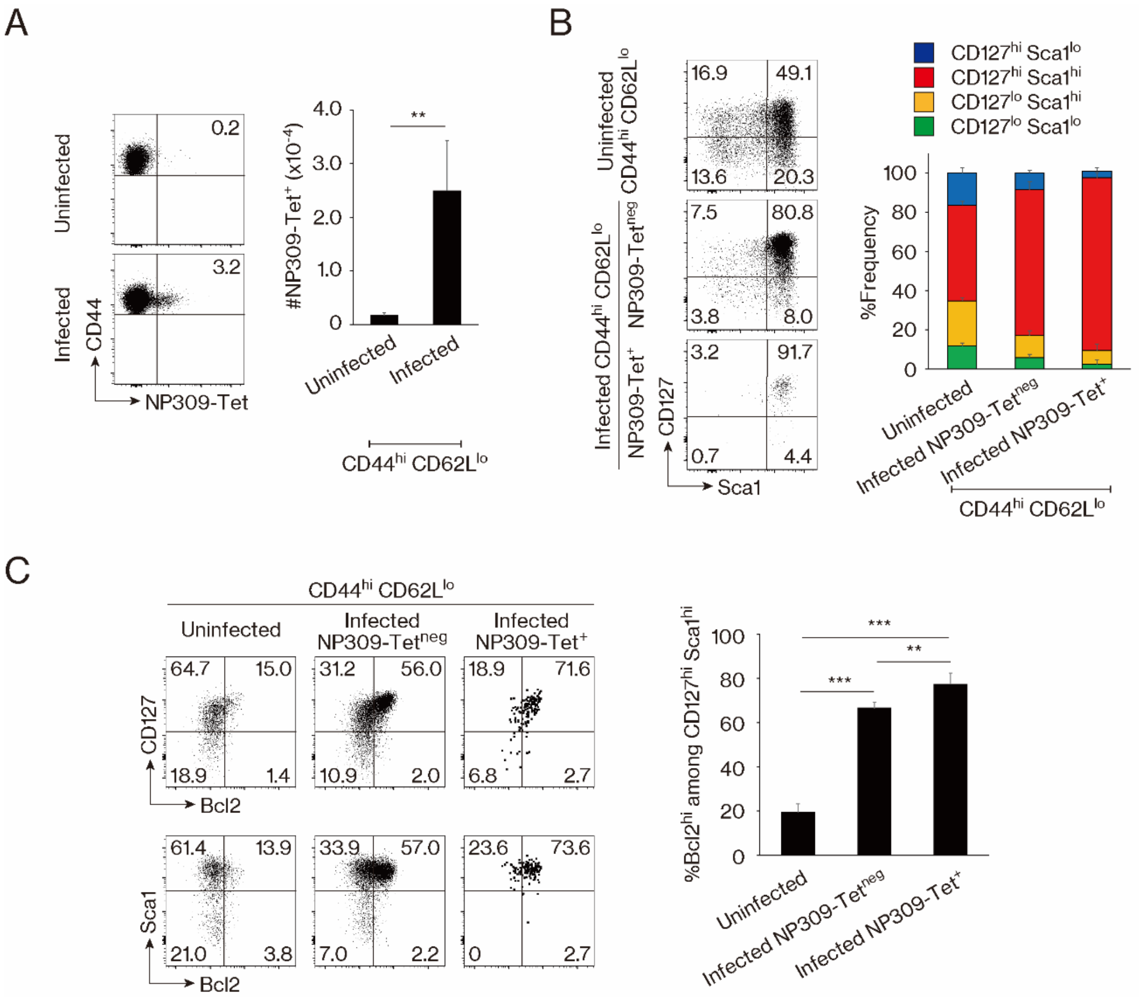

Figure S1. NP309-specific memory CD4$^+$ T lymphocytes have a CD127^{hi} Sca1^{hi} Bcl2^{hi} phenotype. (A) The representative dot plots show NP309 tetramer binding in CD44^{hi} CD62L^{lo} CD4$^+$ T lymphocytes from uninfected versus LCMV-infected mice while the bar graph indicates the number (mean ± S.D.) of NP309-tetramer$^+$ cells in each group (n=5 mice). **(B)** Dot plots showing CD127 and Sca1 expression in the indicated cell populations as well as a bar graph depicting the frequency (mean ± S.D.) of each subpopulation among the same populations are displayed (n=5 mice). **(C)** The representative dot plots show CD127, Sca1, and Bcl2 expression in the indicated cell populations from uninfected and LCMV-infected animals while the bar graph indicates the frequency (mean ± S.D.) of Bcl2^{hi} fraction among CD127^{hi} Sca1^{hi} cells from the same populations (n=3-4 mice). Data are representative of 2 independent experiments performed. ** p<0.01, *** p<0.001.

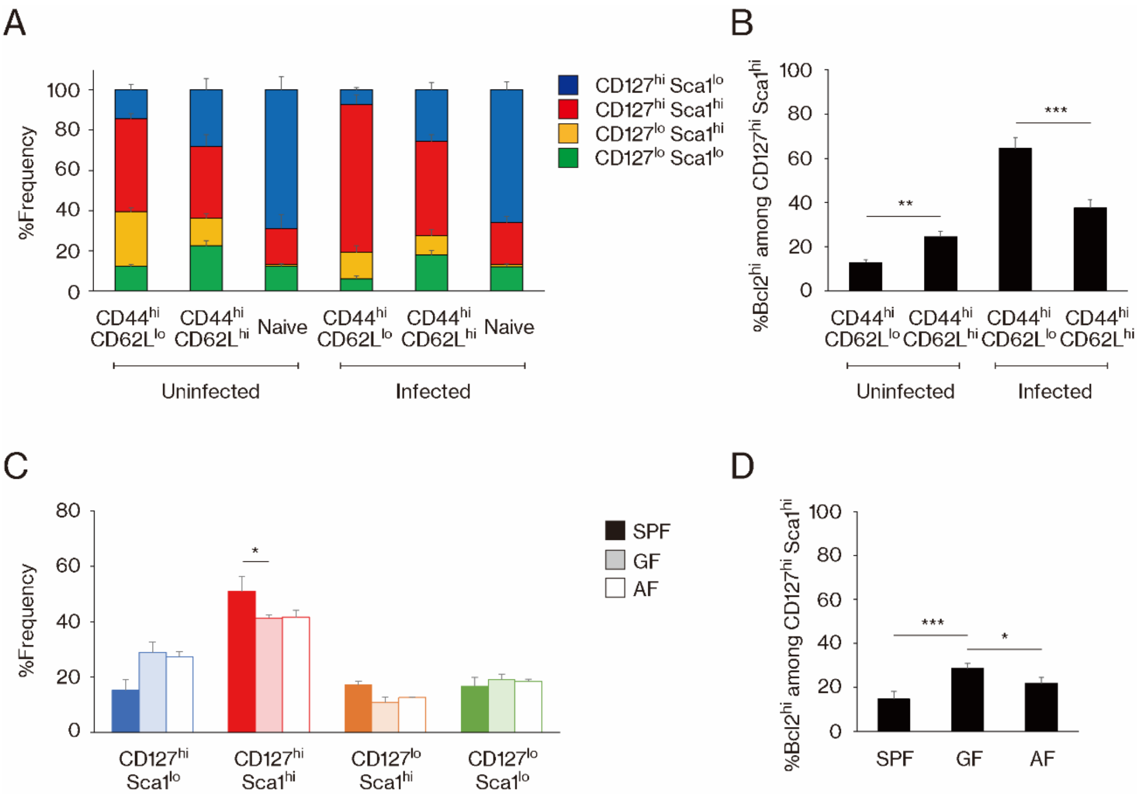

Figure S2. CD44^{hi} CD62L^{hi} CD4^{+} T lymphocytes in uninfected SPF, GF, AF, and LCMV-infected mice. (A) The bar graph shows the frequency (mean ± S.D.) of CD127^{hi} Sca1^{lo}, CD127^{hi} Sca1^{hi}, CD127^{lo} Sca1^{hi}, and CD127^{lo} Sca1^{lo} cells among the indicated CD4^{+} T cell populations from uninfected SPF and infected mice (n=5 mice). **(B)** The bar graph indicates the Bcl2^{hi} fraction (mean ± S.D.) among CD127^{hi} Sca1^{hi} cells from CD44^{hi} CD62L^{lo} and CD44^{hi} CD62L^{hi} CD4^{+} T cells in uninfected and LCMV-infected mice (n=3-4 mice). **(C)** The frequency (mean ± S.D.) of the indicated subsets among total CD44^{hi} CD62L^{hi} CD4^{+} T lymphocytes from SPF, GF, and AF mice (n=3-4 mice). **(D)** The Bcl2^{hi} fraction (mean ± S.D.) among CD127^{hi} Sca1^{hi} CD44^{hi} CD62L^{hi} CD4^{+} T cells from SPF, GF, and AF animals (n=3-4 mice). Data are representative of 2 independent experiments performed. * p<0.05, ** p<0.01, *** p<0.001.

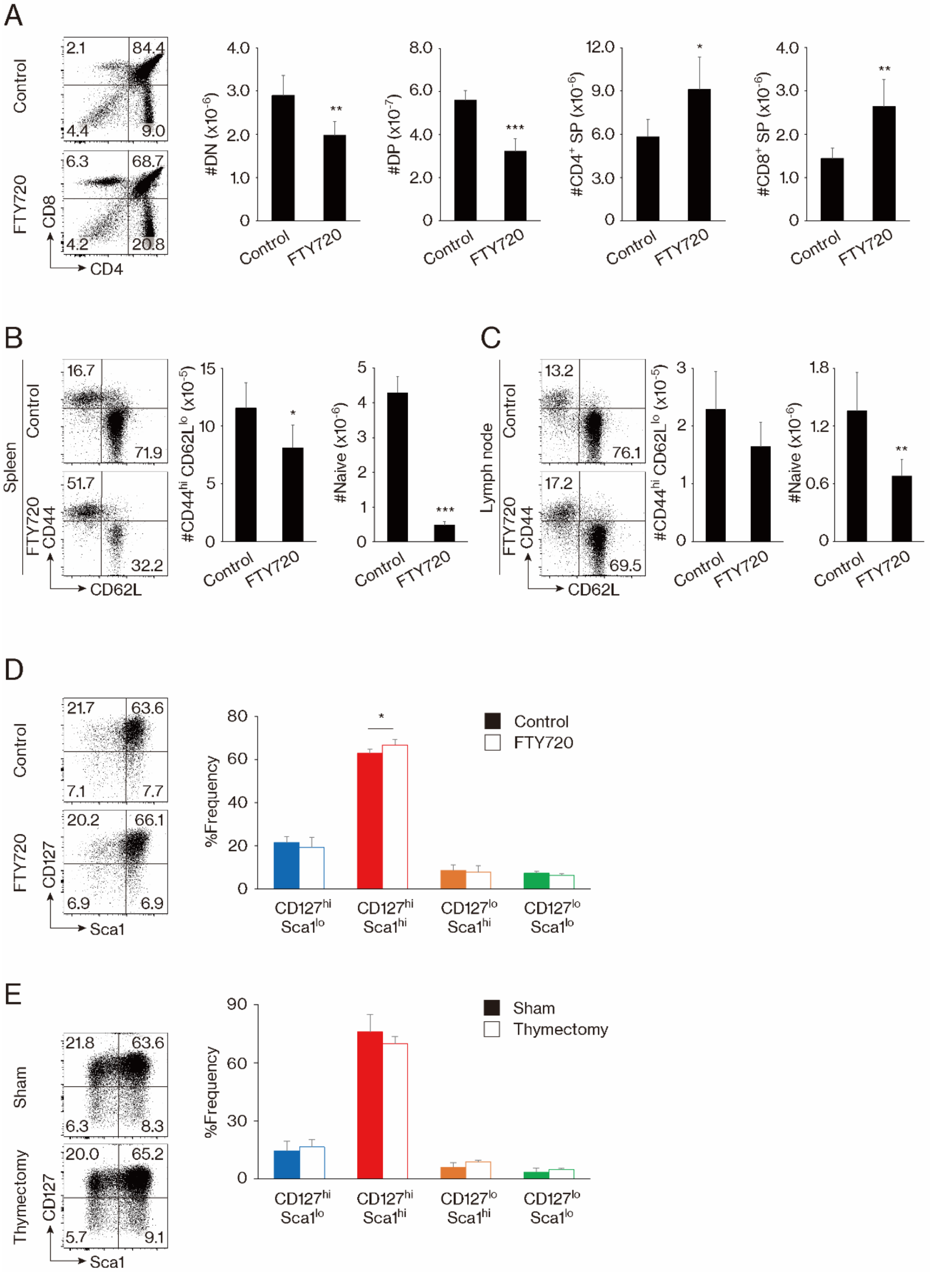

Figure S3. The four MP subpopulations in the periphery are maintained in the absence of thymic input. (A – D) Mice received FTY720 or control PBS and were

analyzed 2 weeks later. (A) Dot plots showing CD4 and CD8 expression in thymocytes from each group together with bar graphs indicating the number (mean ± S.D.) of double-negative (DN), double-positive (DP), and $CD4^+$ and $CD8^+$ single-positive (SP) thymocytes (n=5 mice). (B, C) Dot plots displaying CD44 and CD62L expression in $CD4^+$ T lymphocytes in the (B) spleen and (C) lymph nodes as well as bar graphs depicting the number (mean ± S.D.) of MP and naïve cells from each group (n=5 mice). (D) Dot plots showing CD127 and Sca1 levels in splenic MP $CD4^+$ T lymphocytes from each group and a bar graph indicating the frequency (mean ± S.D.) of MP subpopulations among the total MP population (n=5 mice). Data are representative of 2 independent experiments. **(E)** Mice underwent adult-thymectomy or sham-operation and were analyzed for their MP T lymphocytes 2 weeks later. The representative dot plots display CD127 and Sca1 expression in MP cells from each group while the bar graph indicates the frequency (mean ± S.D.) of MP subpopulations among total MP cells (n=4 mice). Data are pooled from 3 independent experiments performed. * $p<0.05$, ** $p<0.01$, *** $p<0.001$.

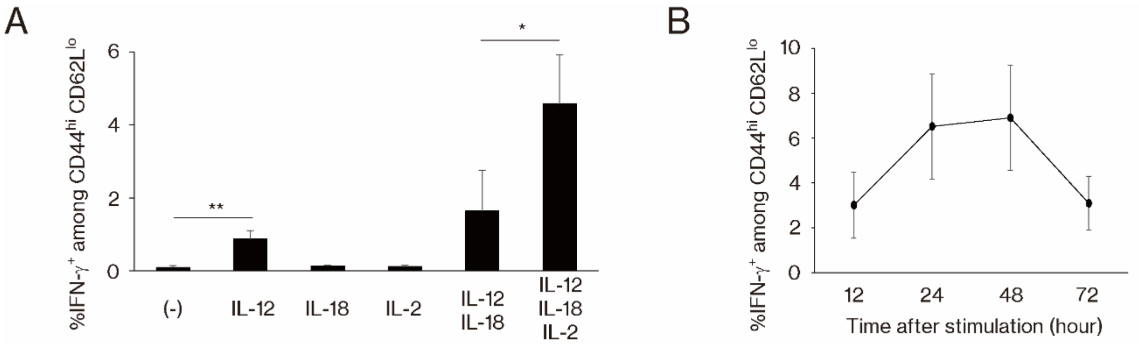

Figure S4. MP cells respond to IL-12, IL-18, and IL-2 and produce IFN-γ. Total splenocytes were cultured **(A)** under the indicated conditions for 24 hours or **(B)** in the presence of IL-12, IL-18, and IL-2 for the indicated period. The graphs indicate the frequency (mean ± S.D.) of IFN-γ^+ cells among MP $CD4^+$ T lymphocytes (n=4 mice). Data are representative of 2 independent experiments. * p<0.05, ** p<0.01.

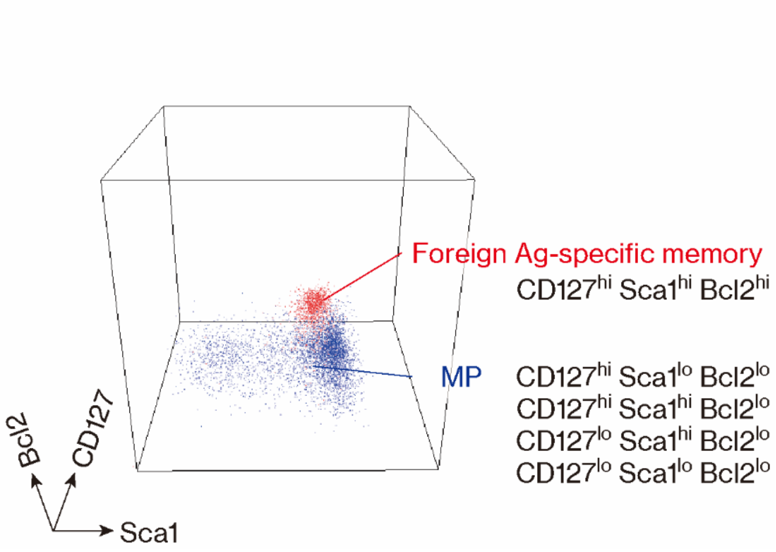

Figure S5. Foreign Ag-specific and MP CD4^{+} T cells represent phenotypically distinct populations. Foreign Ag-specific memory cells are CD127^{hi} Sca1^{hi} Bcl2^{hi} (red) while MP cells are CD127$^{lo\sim hi}$ Sca1$^{lo\sim hi}$ Bcl2^{lo} (blue). Among the latter, CD127^{hi} Sca1^{hi} cells represent the most mature MP subset with the Th1-type inflammatory potential.

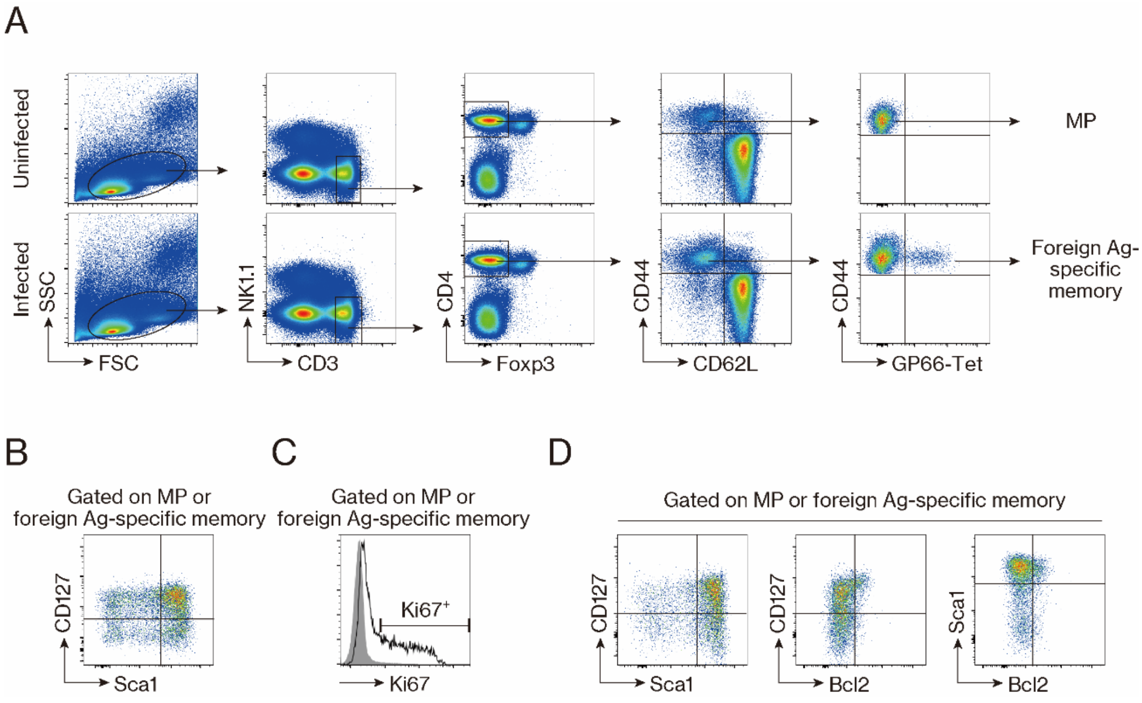

Figure S6. Gating strategy for flow cytometric analyses. (A) To detect MP and foreign Ag-specific memory CD4^{+} T cells, total singlet cells were gated for CD3^{+} NK1.1^{neg} CD4^{+} Foxp3^{neg} CD44^{hi} CD62L^{lo} population. MP and foreign Ag-specific memory cells were then defined as tetramer^{neg} cells from uninfected mice and tetramer^{+} cells from LCMV-infected mice, respectively. **(B)** To analyze MP subsets, CD127 and Sca1 expression levels were measured in MP or foreign Ag-specific memory cells determined in (A). **(C)** For examination of proliferation status, Ki67 levels were measured in MP subsets as well as foreign Ag-specific memory cells using naïve cell population as a reference. **(D)** For detection of Bcl2, CD127, Sca1, and Bcl2 levels were simultaneously analyzed in MP and foreign Ag-specific memory cells. Bcl2^{hi} fraction was determined as the frequency of Bcl2^{hi} cells among CD127^{hi} Sca1^{hi} MP or foreign Ag-specific memory T lymphocyte populations.

Cell cycle (positive regulators)	Cell cycle (negative regulators)	Anti-apoptosis
Aurka	*Cdkn1a*	*Bcl2*
Aurkb	*Cdkn1b*	*Bcl2a1*
Ccna2	*Cdkn2a*	*Bcl2l1*
Ccnb1	*Cdkn2b*	*Bcl2l2*
Ccnb2	*Cdkn2c*	*Bcl2l10*
Ccnd1	*Cdkn2d*	*Mcl1*
Ccnd2	*Chek1*	*Naip1*
Ccnd3	*Chek2*	*Naip2*
Ccne1	*E2f4*	*Naip3*
Cdc25a	*E2f5*	*Naip4*
Cdc25b	*E2f6*	*Naip5*
Cdc25c	*E2f7*	*Naip6*
Cdk1	*E2f8*	*Naip7*
Cdk2	*Rb1*	
Cdk4	*Trp53*	
Cdk6	*Wee1*	
E2f1		
E2f2		
E2f3		
Mdm2		
Plk1		
Plk3		
Plk4		
Tfdp1		
Tfdp2		

Table S1. T cell gene signatures.
